# Conservative treatment of Angle Class III malocclusion with anterior crossbite

**DOI:** 10.1590/2176-9451.20.4.091-098.bbo

**Published:** 2015

**Authors:** João Hélder Ferreira de Aguiar

**Affiliations:** 1 Specialist in Orthodontics, Associação Brasileira de Ortodontia (ABO/EAP), Alfenas, Minas Gerais, Brazil

**Keywords:** Angle Class III malocclusion, Crossbite, Corrective orthodontics

## Abstract

Angle Class III malocclusion is characterized by anteroposterior dental discrepancy which might be associated or not with skeletal changes. Class III molar relationship is associated with vertical or lingually tipped mandibular incisors and a usually concave profile. These characteristics seriously affect facial esthetics and most frequently are the reason why patients seek orthodontic treatment. This case was presented to the committee of the Brazilian Board of Orthodontics and Facial Orthopedics (BBO) as part of the requisites to become a BBO Diplomate.

## Abstract (pt)

A má oclusão de Classe III de Angle é caracterizada por uma discrepância dentária anteroposterior, que pode ou não estar acompanhada por alterações esqueléticas. Observa-se uma relação molar de Classe III associada ao posicionamento vertical ou retroinclinado dos incisivos inferiores e, geralmente, perfil facial côncavo. Esse aspecto gera grande comprometimento estético na face, sendo justamente esse o fator que, na maioria das vezes, motiva o paciente a procurar pelo tratamento ortodôntico. O presente caso clínico foi apresentado à Diretoria do Board Brasileiro de Ortodontia e Ortopedia Facial (BBO) como parte dos requisitos para a obtenção do título de Diplomado pelo BBO.

## INTRODUCTION

A 15 year and seven-month-old, Caucasian, male patient presented for clinical examination with the chief complaint of not being happy with the results of a four-year treatment with removable appliances and that he felt that his chin was pronouncedly placed forward. His medical and dental history was not significant,^2^ and he reported no parafunctional habits. 

## DIAGNOSIS

**Figure 1.**
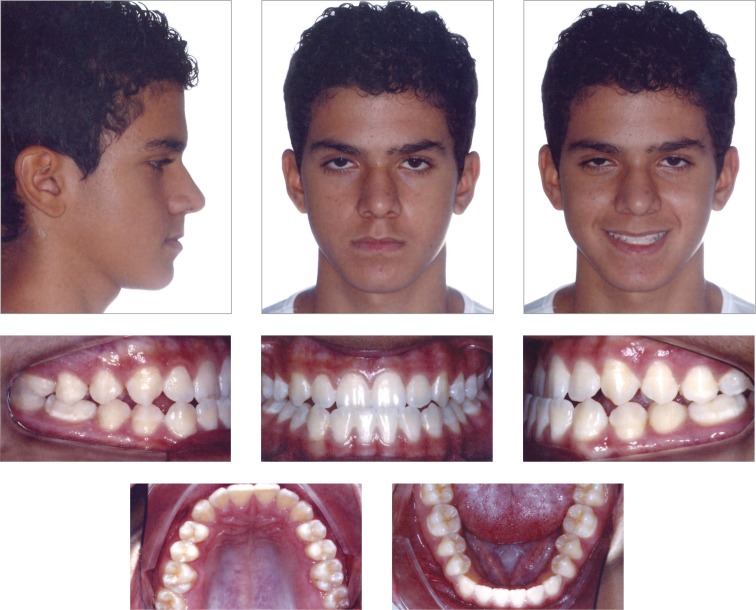
Initial facial and intraoral photographs.

**Figure 2.**
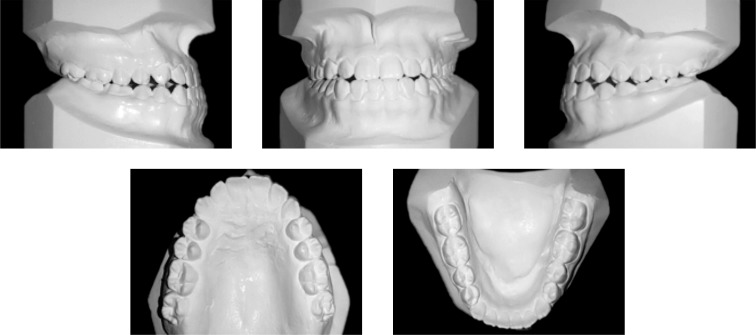
Initial casts.

Extraoral clinical examination revealed symmetric facial pattern and lip competence, as well as lower lip protrusion in relation to the upper lip. The lateral view showed a slightly concave profile (Fig 1). Dental evaluation revealed anterior crossbite which negatively affected facial esthetics. The patient had Angle Class III malocclusion, leveled curve of Spee, normal overbite and, due to anterior crossbite, negative overjet (Figs 1, 2).

**Figure 3.**
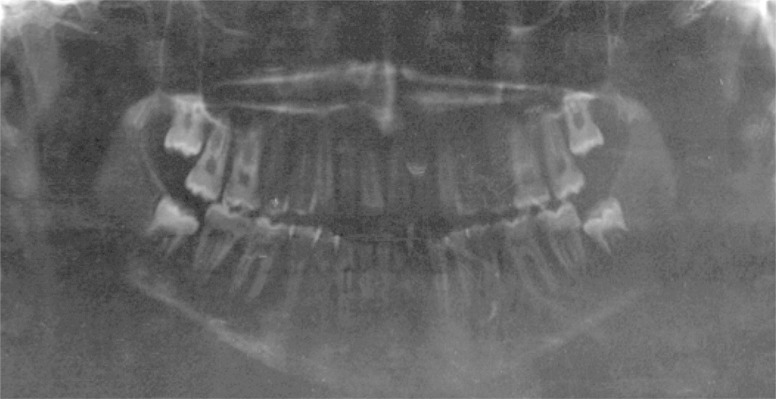
Initial panoramic radiograph.

**Figure 4.**
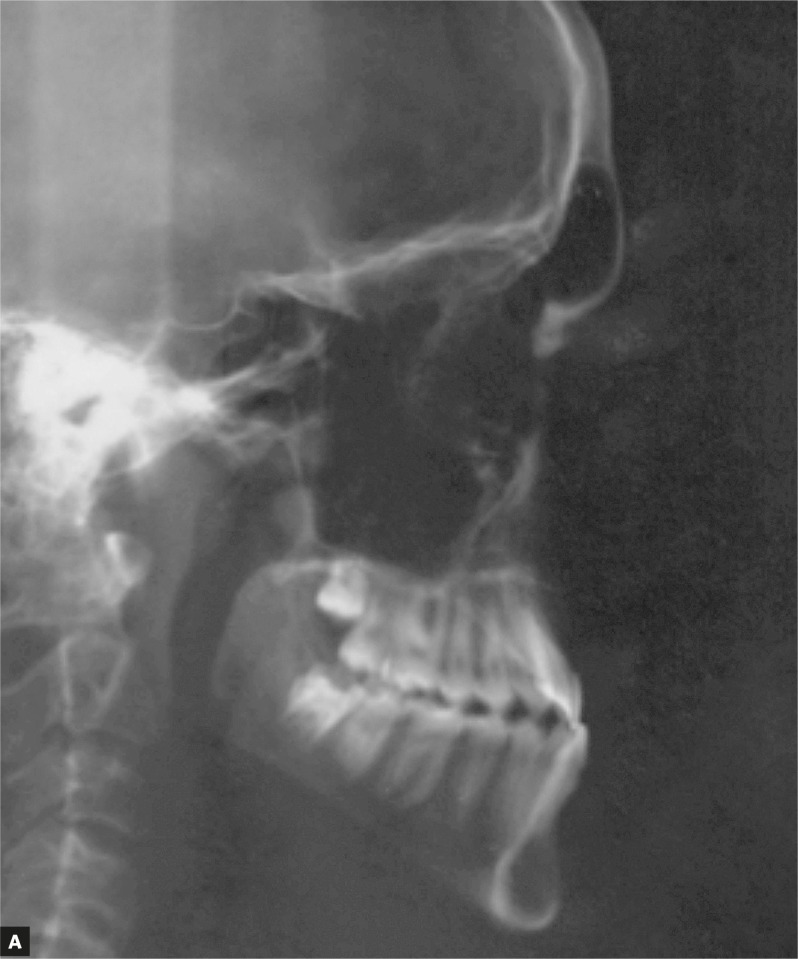
Initial cephalometric profile radiograph (A) and cephalometric tracing (B).

Initial panoramic radiograph showed that the patient had all permanent teeth, including third molars, and that the outlines of tooth roots and alveolar bone were normal (Fig 3). Cephalometrically, (Fig 4 and Table 1) examinations revealed a Class III skeletal pattern (ANB = -2^o)^, with a slightly retruded maxilla in relation to the skull base, and a protruded mandible in relation to both (SNA = 84^o^ and SNB = 86^o)^. His growth pattern was balanced, with slight tendency towards predominantly vertical growth pattern (SN-GoGn = 34^o^, FMA = 29^o^ and Y-axis = 63^o)^. Moreover, the position of maxillary incisors was satisfactory (1-NA = 23^o ^and 5 mm), and his mandibular incisors were inclined lingually (1-NB = 16^o^ and 4 mm). Functional analysis of tongue position did not reveal any changes during mastication, deglutition or speech.

**Table 1.** Initial (A) and final (B) cephalometric values.

	Measurements		Normal	A	B	Dif. A/B
Skeletal pattern	SNA	(Steiner)	82°	84^o^	86^o^	2
	SNB	(Steiner)	80°	86^o^	88^o^	2
	ANB	(Steiner)	2°	-2^o^	-2^o^	0
	Angle of convexity	(Downs)	0°	-8^o^	-3^o^	5
	Y-axis	(Downs)	59°	63^o^	61^o^	2
	Facial angle	(Downs)	87°	88^o^	89^o^	1
	SN-GoGn	(Steiner)	32°	34^o^	33^o^	1
	FMA	(Tweed)	25°	29^o^	24^o^	5
Dental pattern	IMPA	(Tweed)	90°	77^o^	81^o^	4
	1.NA (degrees)	(Steiner)	22°	23^o^	46^o^	23
	1-NA (mm)	(Steiner)	4 mm	5 mm	10 mm	7
	1.NB (degrees)	(Steiner)	25°	16^o^	21^o^	5
	1-NB (mm)	(Steiner)	4 mm	4 mm	5 mm	1
	- Interincisal angle	(Downs)	130°	142^o^	123^o^	19
	1-APo	(Ricketts)	1 mm	4 mm	5 mm	1
Profile	Upper lip — S-line	(Steiner)	0 mm	-2 mm	-1 mm	1
	Lower lip — S-line	(Steiner)	0 mm	0 mm	1 mm	1

## TREATMENT PLAN

Although occlusal findings suggested the need for a more invasive treatment approach because of negative overjet in the anterior region, patient's age and facial esthetics pointed towards conservative treatment. Therefore, treatment was planned to correct Class III skeletal pattern by taking advantage of his remaining growth potential and by performing tooth movement to achieve buccal inclination of maxillary incisors.^3^

A decision was then made to choose mechanisms that would facilitate, during alignment and leveling phases, the achievement of normal occlusion between molars and between canines, correct lateral and protrusive guidance and achieve inclination of maxillary incisors, so as to obtain greater projection of the upper lip and preserve passive lip seal. To this end, the use of fixed orthodontic appliance was chosen for both maxillary and mandibular arches. The retention phase, to be initiated after the completion of active treatment, comprised the use of a modified removable Hawley retainer for the maxillary arch, indicated to be used for 14 to 18 hours a day for one year, followed by overnight use for two more years. A fixed intercanine bar made of 0.016-in untampered stainless steel was placed in the mandibular arch. In that phase, special attention should be given to the development of third molars.

## TREATMENT PROGRESSION

Orthodontic treatment was carried out with Edgewise appliance (Rickets, 0.018 x 0.030-in slot) for the maxillary and mandibular arches.^4^Initially, double-tube bands were placed on teeth #16 and #26, followed by teeth #17 and #27, and bonding of the other teeth in the maxilla. A Blue Elgiloy 0.016 x 0.016-in multiloop archwire was used for alignment and leveling; all lateral segments were aligned by multiloops segmented archwires and nickel-titanium overlay (0.012, 0.014 and 0.016-in). Treatment was completed with the use of a Blue Elgiloy 0.016 x 0.022-in wire of ideal shape and torque. During the active phase of treatment, Class II intermaxillary elastics were used.

 In the mandible, the fixed appliance was also initially placed in posterior teeth. Double-tube bands were placed on teeth #36 and #46, followed by teeth #37 and #47, after which bonding of the other teeth was carried out. A 0.016 x 0.016-in mandibular multiloop archwire was used for alignment and leveling, whereas 0.016 x 0.016-in segmented multiloop archwires were used for alignment of lateral segments, and a 0.016 x 0.022-in archwire of ideal shape and torque was used in association with the mandibular arch for treatment completion.

In the intermediate phase of treatment, leveling was completed with continuous archwires with L-shaped loops placed between lateral incisors and canines on both sides in the maxillary arch. During this phase, Class III intermaxillary elastics were used first in Kobayashi ligatures tied to teeth #33 and #43, and later in L-loops attached to the mandibular arch.

As planned, once treatment objectives were achieved, a removable modified Hawley retainer was used for retention in the maxillary arch for one year, with an indication of use of 14 to 18 hours a day, followed by overnight use for the next two years. After that, the appliance was used every other night for four months and once a week for another four months, after which its use was discontinued. In the mandibular arch, an untampered stainless steel 0.016-in intercanine bar was placed after final dental examinations were carried out.

## RESULTS

**Figure 5.**
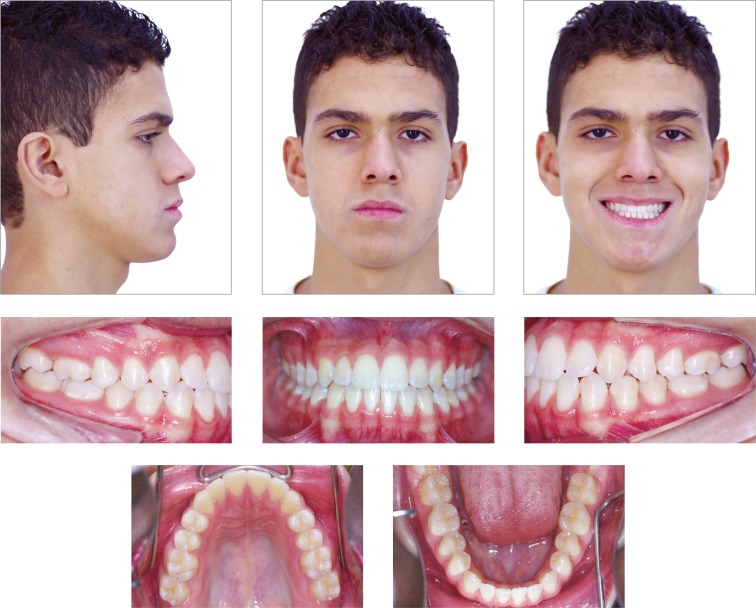
Final facial and intraoral photographs.

**Figure 6.**
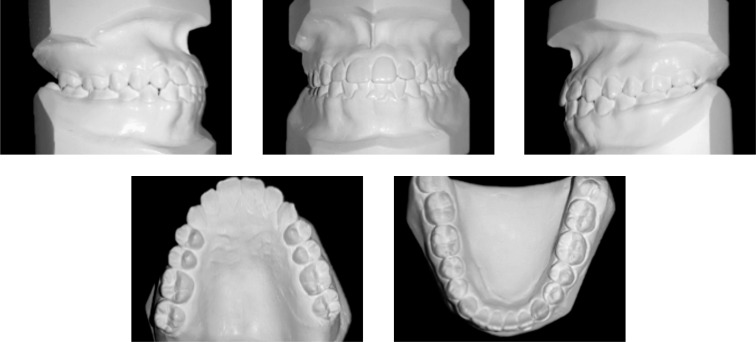
Final casts.

**Figure 7.**
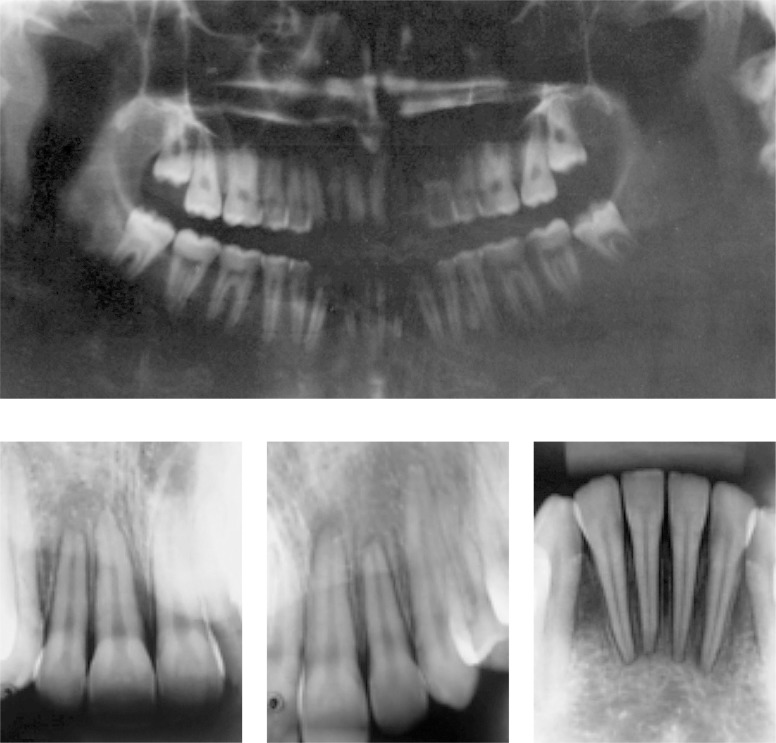
Final panoramic and periapical radiographs of maxillary and mandibular incisors.

**Figure 8.**
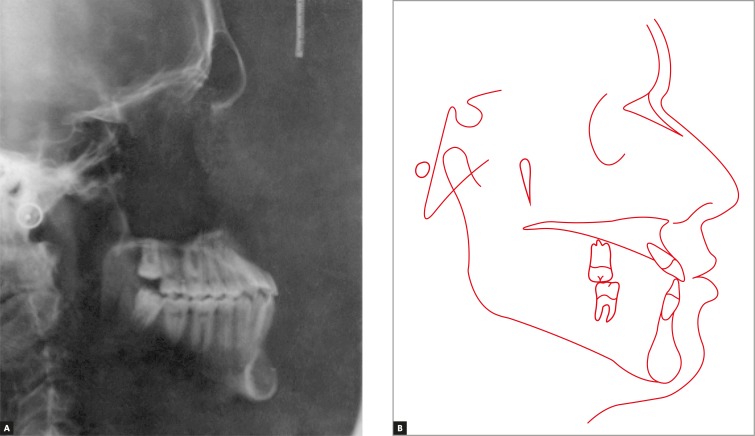
Final cephalometric profile radiograph (A) and cephalometric tracing (B).

**Figure 9.**
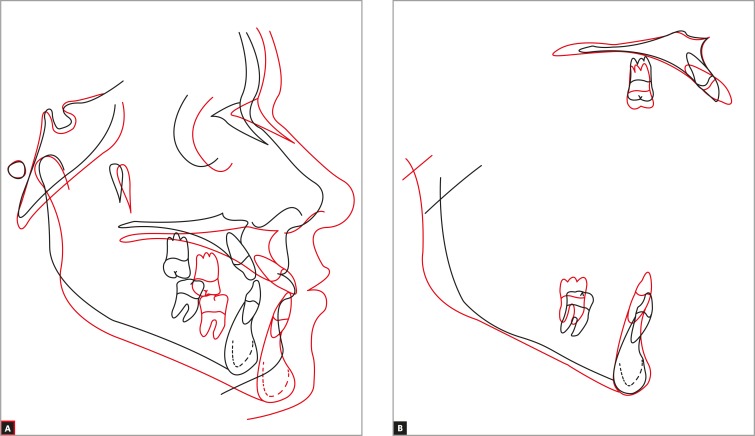
Total (A) and partial (B) superimposition of initial (black) and final (red) cephalometric tracings.

After one year and seven months, the achievement of all objectives was confirmed during final examinations (Figs 5 to 9). Facial esthetics was satisfactory because of the harmonious forward and downward growth of the face, both in the middle third and in the lower third of the face. Moreover, there was substantial change in exposure of maxillary incisors at smiling, as well as in smile esthetics in the composition of facial beauty.

Normal occlusion of molars and canines was achieved, as well as adequate lateral and protrusive guided occlusion. The anteroposterior relationship between maxilla and mandible remained unchanged, as ANB remained at -2^o^. Therefore, the projection of maxillary incisors resulted in adequate anterior crossbite and overjet, as planned. Table 1 shows that 1-NA went from 23^o^ and 5 mm to 46^o ^and 10 mm.

Radiographic examination showed minor root resorption in the apical third of teeth #12 and #22, compatible with orthodontic treatment, probably due to buccal movement of those teeth. Superimposition of initial and final cephalometric tracings revealed substantial maxillary and mandibular growth forward and downward, associated with displacement of the middle and lower facial thirds in the same direction. Partial maxillary superimposition showed changes in the axial inclination of maxillary incisors, as well as their degree of protrusion. Partial mandibular superimposition showed compensatory alveolar growth of both incisors and molars, as well as maxillary molars.

## FINAL CONSIDERATIONS

Angle Class III malocclusion may include several dental and skeletal variations. After identification and definition of their severity, orthodontists should plan adequate treatment by analyzing the advantages and disadvantages of all therapeutic options. Treatment should be initiated with the purpose of correcting abnormalities and should always be based on data about patient's history, facial assessment and reliable and complete cephalometric analyses.

In the case described herein, the projection of maxillary incisors was of paramount importance to obtain adequate overjet. The beginning of treatment was key to its success, as substantial facial growth was identified. Treatment objectives were fully achieved, and function, health and esthetics are adequate.
